# Basalt Fibers Reinforced Concrete: Strength and Failure Modes

**DOI:** 10.3390/ma15207350

**Published:** 2022-10-20

**Authors:** Buthainah Nawaf AL-Kharabsheh, Mohamed Moafak Arbili, Ali Majdi, Saleh M. Alogla, A. Hakamy, Jawad Ahmad, Ahmed Farouk Deifalla

**Affiliations:** 1 Civil Engineering Department, Faculty of Engineering, Al-Albayt University, Al-Mafraq 25113, Jordan; 2 Department of Information Technology, Choman Technical Institute, Erbil Polytechnic University, Erbil 44001, Iraq; 3 Department of Building and Construction Techniques Engineering, Al-Mustaqbal University College, Hillah 51001, Iraq; 4 Department of Civil Engineering, College of Engineering, Qassim University, Buraydah 51452, Saudi Arabia; 5 Department of Physics, Faculty of Applied Science, Umm Al-Qura University, Makkah 21955, Saudi Arabia; 6 Department of Civil Engineering, Military College of Engineering, Sub Campus of National University of Sciences and Technology, Islamabad 44000, Pakistan; 7 Structural Engineering Department, Faculty of Engineering and Technology, Future University in Egypt, New Cairo 11845, Egypt

**Keywords:** basalt fiber, crack prevention, compressive strength, flowability, failure modes

## Abstract

The low tensile capacity of concrete often results in brittle failure without any warning. One way to cope with this issue is to add fibers and essentially improve the tensile strength (TS) behavior of concrete and offset its undesirable brittle failure. In recent investigations, basalt fibers (BFs), as compared to a variety of other kinds of fiber, have attracted the attention of researchers. In that respect, BFs exhibit several benefits, such as excellent elastic properties, great strength, high elastic modulus, higher thermal stability, and decent chemical stability. Although many researchers have reported that BFs can be embedded in concrete to improve the tensile capacity, a more profound understanding of its contribution is still needed. However, the information is scattered and it is difficult for the reader to identify the benefits of BFs. Therefore, a detailed assessment is essential to summarize all relevant information and provide an easy path for the reader. This review (part Ⅰ) summarizes all the relevant information, including flow properties, strength properties, and failure modes. Results reveal that BFs can greatly enhance the strength properties and change the brittle nature of concrete to one of ductility. However, it unfavorably impacts the flowability of concrete. Furthermore, the optimal proportion is shown to be important as a higher dose can adversely affect the strength of concrete, due to a deficiency of flowability. The typical range of the ideal incorporation of BFs varies from 0.5 to 1.5%. Finally, the review also indicates the research gap for future research studies that must be cautiously explored before being used in the real world.

## 1. Introduction 

The most widely used building and construction material worldwide is concrete [1,2,3]. There are, however, also significant issues with concrete materials, including brittleness, poor tensile strength (TS), and capacity to break easily [4,5,6,7]. To strengthen and toughen the concrete, practical and efficient methods must be used. Concrete is reinforced with fibers to increase its TS, flexural strength (FS), toughness, impact resistance, fatigue resistance, abrasion resistance, and ductility [8,9,10]. Different types of fibers are used to improve the tensile capacity of concrete [11,12,13,14]. The kinds of fibers, their geometries, orientations, and densities affect the concrete’s qualities. In recent years, it has been discovered that basalt fibers (BFs) in concrete have superior characteristics and are more affordable than other fibers [15,16]. Although BF-reinforced concrete provides multiple benefits including high tensile strength and elastic modulus, more resistance to acid also improves its strength. On the other hand, it also has some disadvantages, such as reduced flow properties, and the price of BFs being higher than those of E-glass fibers. However, the advantages of BFs are much greater than the disadvantages, which attracts the attention of researchers.

By changing the traditional and energy-intensive techniques of using steel reinforcing bars and wire mesh with fibers, building costs may be reduced overall [17,18,19]. Therefore, labor expenses will be cheaper, as will upkeep expenses, the amount of time needed for construction, and the overall cost of the building. Energy will also be conserved, since the quantity of fiber used is frequently considerably less than the raw materials required to produce traditional reinforcement. Glass fibers are produced using the spinneret technique, which is comparable to the manufacture of continuous BFs [20]. The melt is often heated using overhead gas burners while making glass fibers. The melting point of glass is 1400–1600 °C, which means that glass fibers work better than basalt when subjected to acids but are less resistant to alkalis than BFs [21]. Because glass has poor alkali resistance, its main disadvantage is that it deteriorates in alkaline environments [17]. Concrete has a limited range of applications because of its alkaline environment.

The drawbacks of steel fibers include reduced workability, and corrosion. Although the addition of glass fibers makes concrete more resilient, the alkali assault reduces concrete’s long-term strength. One disadvantage of the more rigid and powerful carbon fibers is their high price. The melting temperature and elastic modulus of nylon and polypropylene fibers are both low [22]. 

In terms of strength, elasticity, and load transfer associated with surface adhesion qualities, the compatibility of the fiber and matrix has a major impact on the composite’s performance. In several fiber-reinforced composite applications, fibers made of steel, glass, carbon, and polymers are frequently employed. Polymer-based fibers can be used in a variety of ways, and their performance in composites varies greatly [23]. Small repeating units make up the structure of polymers, which are non-metallic compounds [24]. Polymers with a wide range of uses, such as automotive, consumer goods, and home items, have been used in concrete due to their characteristics, such as cheap cost, lightweight, and great corrosion resistance [25]. 

Polymer fibers provide a more desirable material since they are more resistant to alkaline reactions, corrosion, chlorine, and salt [26]. Even at extremely low volume fractions, low modulus of elasticity fibers, such as polymer fiber, are excellent in minimizing cracking during plastic shrinkage. Its usage is generally recognized due to its great efficacy in minimizing cracking during plastic shrinkage [27]. In addition, to reduce plastic shrinkage cracking, polymer fibers have been found to enhance the mechanical characteristics of concrete. Polymer fibers can also be used to strengthen cement-based products under flexural/tensile loads. In this instance, high volume fractions of a minimum of 2 percent of the fibers can be employed in a cement matrix [28]. According to one study, toughness, impact resistance, and fatigue performance have all improved statistically when polypropylene fibers are used in proportions as low as 0.1 percent [29].

In recent years, BFs with non-hazardous and ecologically friendly characteristics have been employed in concrete. These fibers are produced by melting volcanic rocks. BFs are produced using the same technology as glass fiber. However, it is less expensive since it doesn’t include any additives and uses less energy than glass or carbon fibers.

**Figure 1:**
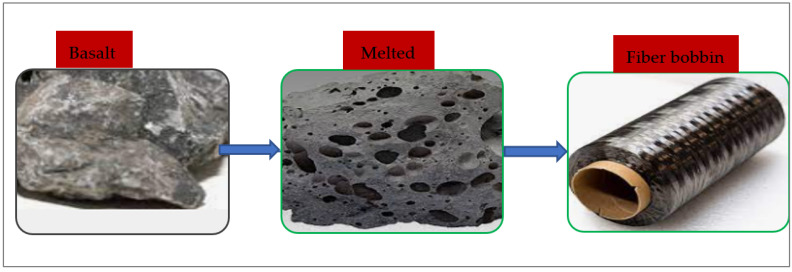
Manufacturing of BFs.

In contrast to other materials such as fiberglass, BFs are manufactured entirely from basalt rock from a carefully selected excavation supply. All that is done is to wash and melt the basalt. It takes melting the mined basalt rock at a temperature of around 1400 °C to produce BFs. A continuous thread of BFs is created by extruding the molten basalt rock via tiny nozzles called spinnerets. The production of BFs is seen in Figure 1.

**Figure 2:**
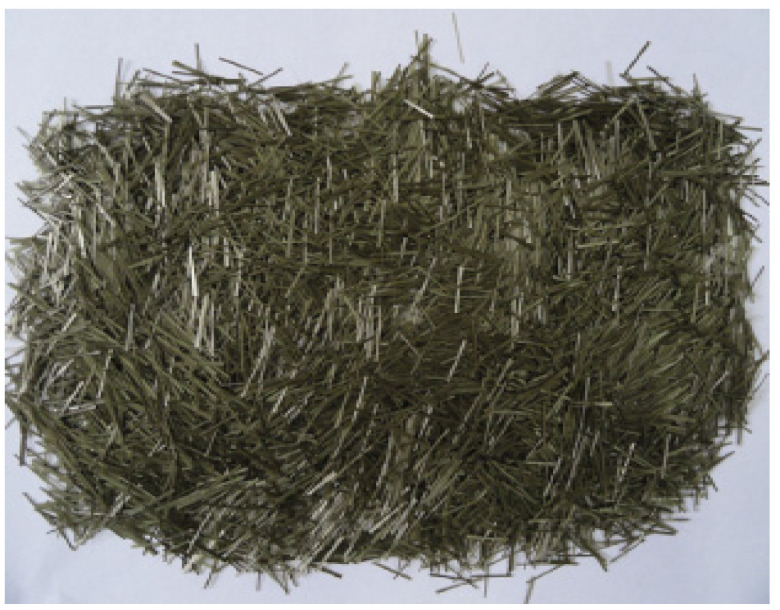
Basalt Fibers [30] with Elsevier permission.

According to Figure 2, most of the BFs used in concrete have a dark brown color. BFs have a TS range of 2800 MPa to 5000 MPa and an elastic modulus range of 90 to 12 GPa. In addition, BFs have an elasticity modulus that is noticeably greater than synthetic fibers but not quite as high as steel. Depending on the source and use, TS and elastic modulus might change. Different physical characteristics of BFs were reported by different scholars. Table 1 outlines the different physical attributes of fibers according to earlier studies.

A brand new type of high-performance, environmentally beneficial, and eco-friendly fiber known as BFs performs very well and is employed extensively across a variety of industries [36]. The purpose of BFs to enhance the mechanical characteristics of regular concrete has recently attracted the interest of numerous scholars, and various suggestions have been documented in the literature [21,37]. However, owing to their superior mechanical qualities, BFs is a rapidly developing material in concrete materials. Compared to PVA fiber, it is much stronger in terms of TS, elastic modulus, and density. In alkaline solution, it exhibits strong chemical stability and corrosion resistance. BFs, a kind of synthetic inorganic fabric manufactured from basalt rock, an igneous rock created from solidified lava, is often used in clothing. Crushed basalt rock is heated to a molten condition at a temperature of around 1400 °C and then passed through tiny nozzles to create continuous BF filaments. The inclusion of BFs means that the mechanical characteristics are enhanced, and they may also be used as reinforcement for concrete, according to preliminary studies [38].

According to research, BFs may greatly increase the hardness and fracture resistance of concrete and have high alkali resistance [39]. The impact of BFs with various volume percentages on the fracture toughness of concrete beams was investigated by one researcher [40]. The ultimate load and deformation of BFs concrete beams rose dramatically before failure, according to their experimental findings, although the susceptibility to fractures was reduced. An investigation [41] into the physical and strength characteristics of cement mortar reinforced with BFs at 28 days showed the ideal BF blending quantity. The mechanical characteristics of self-compacting concrete with BF reinforcement were studied. The results showed that adding BFs makes self-compacting concrete less workable. However, it may significantly enhance the physical characteristics of concrete specimens [42]. An investigation was conducted into the impacts of polypropylene and BFs on mechanical characteristics. The TS and flexural strength (FS) of concrete specimens were said to be greatly enhanced by fiber. However, it is not immediately apparent how compressive strength (CS) strengthens [43]. Results indicated the fiber improved compressive strength by 6% [44]. BFs have excellent mechanical and physical characteristics, including strong corrosion resistance, excellent heat resistance, and resistance to alkalis and acids [45]. According to research, BFs may considerably increase the deformation and energy absorption capabilities of concrete, but they have little to no effect on dynamic CS [46].

Although many researchers reported that BFs can be used in concrete to enhance the tensile strain, the information is scattered and it is difficult for a reader to identify the benefits of BFs in concrete. Therefore, a detailed review is required to summarize all the relevant information and provide an easy path for the reader. This review summarized all the relevant information including fresh properties, strength properties, and failure modes. Results suggest that BFs enhanced the strength properties as did the other fibers. In addition, the failure mode of BFs reinforced concrete is ductile which ensures the safety of a structural member by allowing deformation before failure. However, it unfavorably impacts the flow of concrete. Furthermore, the optimum dose is important as the greater quantity unfavorably impacts the strength properties of concrete due to a deficiency of flowability. The typical range of the ideal amount of BFs fluctuates from 0.5 to 1.5%. Finally, the analysis also indicates s research gap for potential research that must be explored before being used practically. 

## 2. Slump Flow 

**Figure 3:**
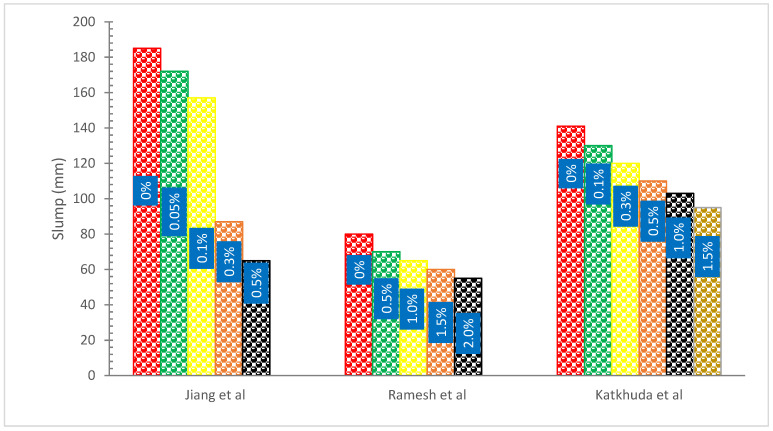
Slump Flow of Concrete: Data source [31,36,50].

Figure 3 shows the slump flow with the addition of basalt fibers. It can be observed that BFs addition decreased the flow of concrete. This may be attributed to the fact that BFS absorbs some moisture and that during mixing, there was more friction between the fibers and cement [31]. Fibers have a bigger surface area, therefore more water is needed to coat them, leaving less water available for lubricating. In addition, the existence of fibers caused the internal friction among the fragments of concrete to rise, necessitating the use of more cement paste [18]. The workability of BFs reinforced concrete diminishes with increasing volume and length of BFs, according to a study [47] that looked at the influence of presoaking BFs on the concrete. The dose of BFs is 3, 5, and 7 kg/m^3^, with the lengths being 10 mm, 20 mm, and 30 mm. BFs absorb some water, reducing workability and droop. In addition, research [48] investigated the effects of different BFs levels on the workability of reinforced concrete. These contents were 0%, 0.10%, 0.20%, 0.30%, 0.40%, and 0.50%. The findings demonstrate that a decrease in slump occurs when the volume percentage of fibers is increased. The workability of the concrete might be reduced because of the BFs. This decrease may be caused by a significant surface area of BFs, which raises the viscosity of new concrete with the addition of BFs, lowering slump. According to prior research [49], fresh concrete with various BFs contents becomes less workable as the fiber content increases.

It was discovered that when the fiber proportion was raised, the slump values of concrete mixtures decreased. When fibers are added at volume fractions of 0.5, 1.0, 1.5, and 2.0 percent, the slump value is reduced to 70 mm, 65 mm, 60 mm, and 55 mm, respectively, from 80 mm for the control mix (BFs 0.0). It may be concluded that fibers added to the concrete mix may be to blame for the decline in workability. The viscosity of concrete may rise as a result of the fiber’s greater surface area needing to absorb much more cement paste [50]. Concrete with a higher BFs content has less slump. The reference concrete has a 185 mm slump. The slumps were decreased to 130, 90, 75, and 59 mm by adding BFs at volume fractions of 0.05, 0.1, 0.15, and 0.2 percent, respectively [51].

According to research [36], the fiber supplementation caused the droop to lessen somewhat. The slump for ordinary concrete is 185 mm. The droop decreases to 142, 124, and 59 mm, respectively, when volume fractions of 0.05, 0.1, and 0.3 percent of polypropylene fibers are added. The droop decreases to 172, 157, 87, and 65 mm, respectively, when BFs with volume fractions of 0.05, 0.1, 0.3, and 0.5 percent are added. It may be inferred that the inclusion of fibers may result in a reduction in fluidity. The dispersed fiber in the concrete, which may create a network structure, is what causes this occurrence because it inhibits the blend from separating and flowing. Because fibers have great content and a big surface area, they may absorb more cement paste to cover them. This causes the blend to become stickier, which lowers the slump [52]. In addition, adding 22 mm BFs to concrete results in a greater slump than adding 12 mm BFs with the same volume percentage. Shorter fibers may have more fibers per unit volume and a higher fiber distribution density, which might be the cause. The slump of the concrete may decrease as a consequence of the increased difficulty the fibers have in dispersing evenly inside the matrix [36]. According to one study [53], fibers with short lengths flow more easily than fibers with longer lengths. Limited-length fibers increase the surface area of the fiber cement paste, improving its ability to bond [54].

## 3. Air Content and Unit Weight 

**Figure 4:**
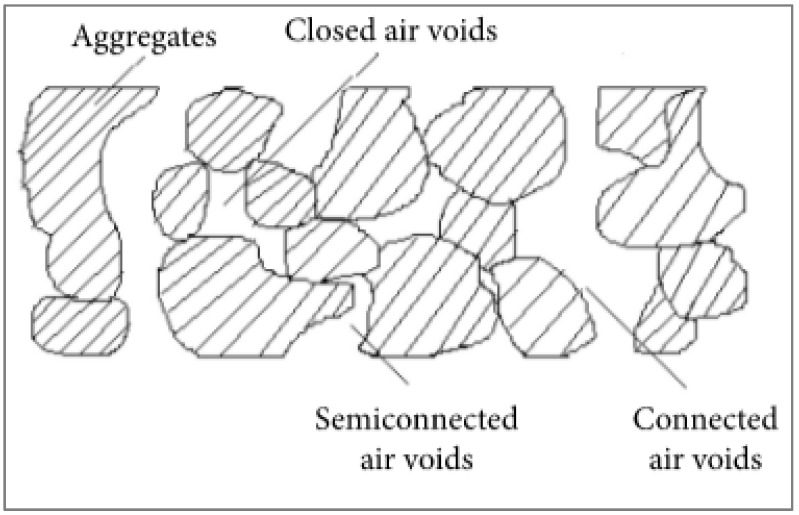
Schematic View of Air Content [55].

There are three different forms of air voids: closed air voids, semi-connected air voids, and linked air voids. According to Figure 4, the linked air voids are the spaces inside the blend that may interact with the outside and serve as drainage and noise absorbers. The strength and durability of concrete are negatively impacted by these gaps.

As a monitoring approach for the volume of the mix, and the air content values, the unit weight of the mixture was used. The air content of the blend, which also impacts the drop of the mix, had an impact on unit weight. The research predicts that when the slump decreases, the air content often rises, which should lower the unit weight value.

**Figure 5:**
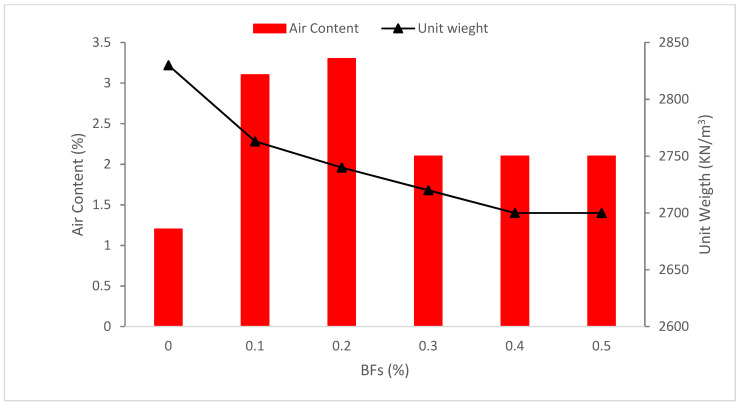
Air Content and Unit Weight: Data Source [61].

Figure 5 displays the air content with the addition of basalt fibers. It can be observed that BFs added increased the air content and decreased the unit weight of concrete. The increased quantity of trapped air has a harmful influence on the strength characteristics of concrete, hence a specific mixing technique to reduce the air content has been developed [56]. According to research, the amount of air in the concrete mix rises as the quantity of polypropylene fibers is boosted. The quantity of cavities and heterogeneity in the concrete mixture are both influenced by the presence of fiber [57].

According to research, the amount of air in a concrete mixture reduced as the quantity of steel fiber in the mixture was boosted, and adding 3 percent of steel fiber resulted in a reduction of entrapped air of between 0.9 percent and 2.4 percent for various water-to-binder ratios. As a result, the effective distribution of short fibers when a greater dose of superplasticizer was applied may explain the lowering impact of entrapped air content in concrete mixes. Other probable factors should be researched in the future [58]. The trials with conventional strength concrete likewise revealed the same diminishing effects of steel fiber on-air content [59]. Other researchers have also seen a rise in the quantity of air in typical concrete, which they ascribe to the challenges long fibers have in being distributed and oriented correctly [60]. Due to the fact that BFs were the lightest element in the concrete mixes, there was a consistent unit weight drop when BFs were added [61]. According to research, the bulk density of the concrete is unaffected by the inclusion of polypropylene fibers [57]. In addition, compared to steel fiber mixes, BFs mixes produce lighter concrete [62].

## 4. Strength Properties 

### 4.1. Compressive Strength (CS) 

**Figure 6:**
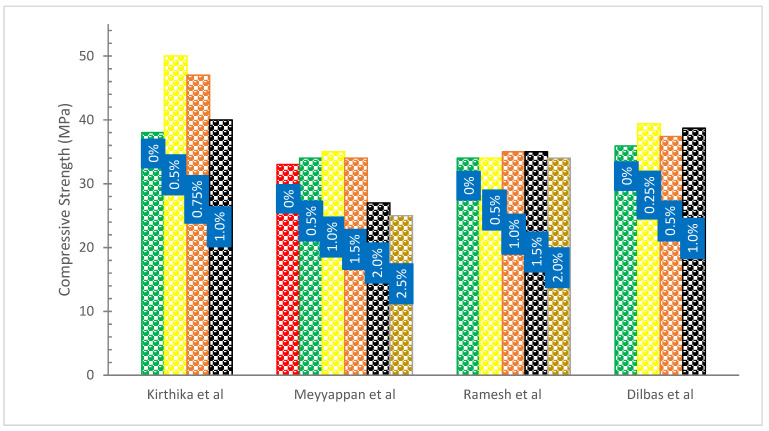
Compressive Strength: Data Source [32,50,63,67].

Figure 6 shows the compressive strength (CS) with the addition of basalt fibers. It can be observed that BF addition increased the CS. When the amount of BFs in the mix was increased, there was a minor improvement in the CS of the material up to 1.5 percent fiber volume. However, when the amount of fiber was increased to 2.0 percent, there was a loss in the CS of the material. The explanation may be because there was a larger quantity of fiber volume fraction in the concrete mix. This may have caused the distribution of fibers to become more uneven, which in turn may have caused a loss in CS. When compared to the conventional concrete matrix, the highest improvements in CS reached 4.45 percent at a fiber volume fraction of concrete that was 1.5 percent [50].

The BFs are added to the concrete in volume fractions of 0.5, 1, 1.5, 2, 2.5, and 3 percent for M-30 grade concrete, which results in seven-day CS that are almost 55 to 64 percent higher than 28-day CS for BFs content of 0 to 3 percent. The CS of ordinary concrete rose by 4.72 percent when BFs were added at a rate of 0.5 percent. The CS rises to 11.45% as compared to reference concrete when BFs are raised to 1%. Because the BFs occupied the concrete pores in a variety of orientations, the strength-improving feature was induced. When BFs concentration is raised further, such as by 1.5 percent, CS decreases to 8.2 percent but increases to 3.45 percent when compared to normal concrete. When BFs concentration exceeds 1.5 percent, the CS gradually decreases, by 16.48 percent, 25.26 percent, and 33.87 percent for BFs contents of 2%, 2.5 percent, and 3%, respectively. This might be the result of the fiber content bunching, problems with compaction, or a lack of bonding between the fibers and concrete elements [63].

According to research [30], the RCA and BF contents have a significant impact on the CS of the concrete. All concrete samples at the age of 90 days have a CS that is at least 20% more than those attained at the age of 28 days, demonstrating that a longer curing time results in a higher strength of RCA. This is probably because recycled aggregate concrete takes longer than regular aggregate concrete to fully hydrate and develop its full CS [64]. The load-bearing capability of composite samples was improved by the inclusion of BFs. The transverse confinement effect and strong connection between the fibers and cement were responsible for this performance improvement [65]. While fibers with 1.0 and 1.5 percent replacement exhibited almost equal CS to control concrete, those with a 0.5 percent replacement had somewhat superior CS than the material. This indicates that the impact of fiber addition on CS is minimal [66].

According to research, adding BFs at 0.5 percent resulted in a 3.9 percent decrease in CS after 28 days [40]. There was no discernible increase in the CS of basalt FRC and polypropylene fiber in this investigation. The CS of the specimens reinforced with polypropylene fiber with a volume fraction of 0.05%, 0.1%, and 3% increases by 2.41 percent, 6.13 percent, and 4.32 percent, respectively, as compared to plain concrete. The BFs exhibited a better CS gain at the same volume fraction when compared to polypropylene fiber. The CS enhancement of reinforced concrete with BFs (12 mm length) varies from 3.74 percent to 6.49 percent when adding v with volume fractions of 0.05, 0.1, 3.0, and 0.5 percent [36].

An adequate quantity of BFs may be included to increase the CS at various ages, according to research [51]. The CS of BFs (0.5%) is the greatest, being 9.87 percent and 17.13 percent greater at 28 and 90 days, respectively, than that of the reference concrete (BFs 0%). The CS does, however, decline as fiber content rises, and the strength decline becomes more pronounced the higher the fiber concentration. The lowest CS is shown by BFs (0.20 percent), which is 8.08 and 5.15 percent less than that of the control concrete after 28 and 90 days, respectively. The three-dimensional randomly dispersed BFs bundles, which are tightly bonded when the fiber content is suitable, limit the transverse deformation under compression [51]. According to the fiber spacing theory, a portion of the load is also carried by the fiber when concrete is loaded because stress expands along the aggregate interface before moving to the fiber position. When the fiber is removed, friction between the fiber and the concrete interface uses up some energy, delaying the concrete’s eventual deterioration. As a result, the CS of the concrete increases. The total fiber surface area rises significantly when the fiber proportion is at a higher ratio. As a result, more cement pastes are needed to coat the fibers, which affects how well the cement pastes and aggregates bond together. In addition, a high fiber content directly reduces the matrix’s average fiber spacing resulting in less strength. Table 2 shows the summary of strength properties of concrete with BF addition.

**Figure 7:**
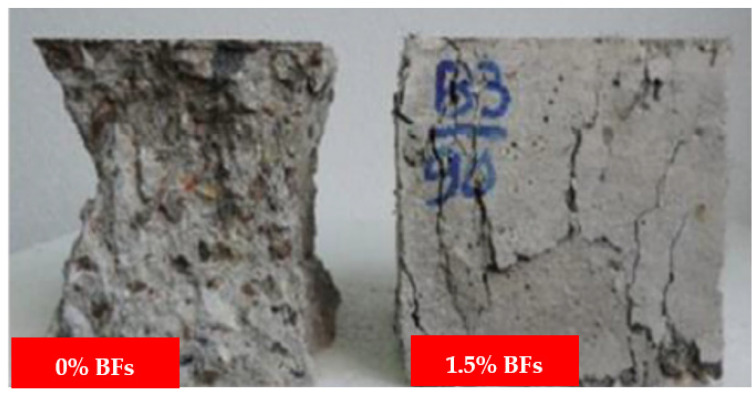
Compressive Failure Modes [50] with Elsevier permission.

The compression test failure mode for cubes is shown in Figure 7. The development of significant fissures over their whole length caused a cube specimen made of plain concrete to collapse. In contrast, adding BFs to the concrete failed because of the formation of small, thin fissures on their surfaces. This demonstrates how well the BFs and concrete matrix adhere to one another. Similar results have been reported by [43]. BF number and dispersion fibers affect the CS of BF concrete. Due to the cyclo hoop effect, the standard concrete cube samples were crushed with a great deal of noise as the weight increased, and Figure 7 illustrates the failure mode of normal concrete. According to the findings of another research study, the fracture in the plain concrete specimen began to form in an angled position place and continued to grow until it finally broke. The destruction mechanism was quick and exhibited brittle qualities in an evident way. 39.8 MPa was determined to be the compression strength value of the plain concrete specimen. The addition of BFs demonstrated that the compression strength value first displayed a small rise, but subsequently fell with the boost in fiber percentage. This pattern continued until the BFs were removed. When the BF percentage was 0.1 percent, the crack breadth shrank but the length remained the same. However, the length did not decrease. Both the breadth and the length of the fracture shrank when the BF content reached 0.2 percent, and many fissures were visible on the surface of the concrete sample at this point. However, the impact of the reinforcement on the compressive capacity was not readily apparent since only a little quantity was used [70].

**Figure 8:**
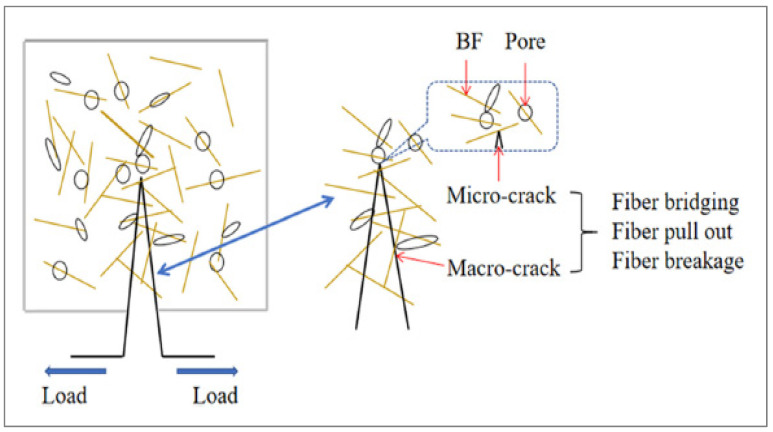
Multi Scale Crack Resistance [73].

Figure 8 illustrates the multi-scale function that BFs play in the process of fracture propagation at both the macro and micro scales. This role is indicative of the toughening and crack-resistant mechanism due to the addition of BFs. After carefully examining the damaged specimen, we were able to establish that it had a fair level of integrity, as shown by the sample’s ability to be broken apart with the assistance of an external force. The specimen also included several winding fractures. In addition, the fracture surface of BF concrete is curved rather than straight, which indicates that the fiber has a clear impact on preventing cracks from occurring.

### 4.2. Tensile Strength (TS)

**Figure 9:**
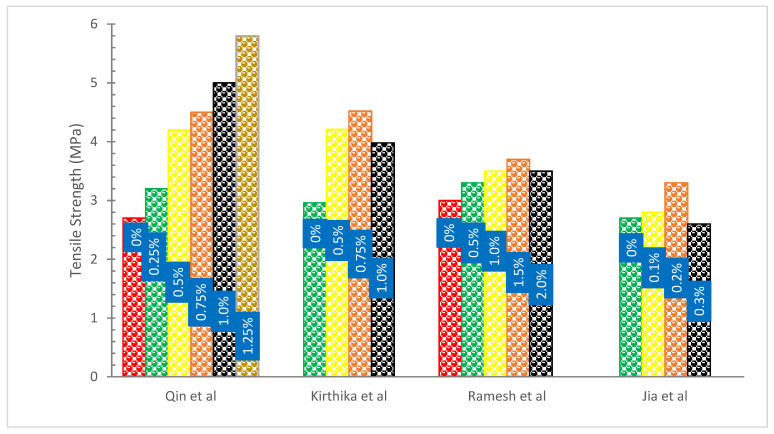
Tensile Strength: Data Source [35,50,67,70].

Figure 9 displays the tensile strength (TS) of concrete with the addition of basalt fibers. It can be observed that the addition of BFs increased the TS of concrete. According to the findings of one study, the performance of BFs in conventional concrete ranged from 0 to 2 percent by volume. The fibers did not have an effect on the CS of the concrete, but they did increase the TS at a content level of 2 percent, which was optimal [74]. As per investigation, the TS of concrete mixes made with BFs was significantly enhanced in comparison to ordinary concrete. When contrasted with the ordinary concrete mix, the BF (1.5%) concrete mix showed a 22.58 percent gain in TS. In comparison to the reference concrete, the TS of concrete mixes with a BF content of 0.5%,1.0%, 1.5%, and 2.0% were found to be improved by 9.6,12.90, 22.58, and 16.12%, respectively. Due to the bridging action of the fibers across the fractures, which effectively inhibits the development of cracks, it may contribute to a boost in the strength of fiber concrete specimens. Based on the findings, the conclusion can be drawn that a raise in the proportion of BFs results in a rise in the TS of the concrete. The quantity of BFs and dispersion fibers used in BF concrete directly affects the material’s TS [50]. It was discovered that the use of chopped BFs had a substantial impact on the TS of the concrete. These results may be attributed to the composition of the BFs as well as the high TS of the fiber, which reached a value of 4840 MPa [31].

A study was conducted by a researcher to investigate the effects on the properties and strength of concrete caused by the addition of BFs of varying lengths. The author carried out research on the effect that employing different BFs lengths of 10, 20, and 30 mm had on the TS of concrete, and he published his findings. According to the findings, the BFs with a diameter of 30 mm have the potential to provide the greatest TS [47]. However, according to the findings of the research, increasing the length of the BFs from 6 to 30 mm generated a maximum drop in splitting TS of 13 percent. This was found when the fiber content was kept at 0.5 percent. In addition, it is important to point out that the composites made with BFs had a greater seven-day TS than the composites made with glass fibers. This may be attributed to the increased TS of the BFs that was used [35].

When compared to plain concrete, the TS of concrete seems to improve with an increase in fiber percentage. This contrasts with the plain concrete. The splitting TS shown by the BF concrete is much greater than that of the concrete reinforced with polypropylene fibers. The TS of concrete reinforced with BFs of12 mm improves by about 14.08–24.34 percent when the fiber content of the concrete is increased. In addition, an increase of around 14.96–25.51 percent may be seen in the splitting TS of BFs (22 mm) reinforced concrete [36]. 

The TS of high-performance concrete (HPC) reinforced with single BFs increased by 21.90 percent –38.33 percent when compared to the TS of HPC without fiber. Meanwhile, the TS of HPC reinforced with single polypropylene fibers increased by 32.86 percent–44.52 percent. The findings indicate that the incorporation of BFs or polypropylene fiber into concrete results in the formation of a three-dimensional chaotic distribution within the material. This, in turn, can delay the onset of cracking and lead to an increase in the splitting TS of high-performance concrete (HPC). According to the results of the correlation research, polypropylene fibers that have a low elastic modulus have a significant impact on the splitting TS of HPC [43].

According to the findings of the research, the incorporation of polypropylene fiber and carbon fiber into concrete at a volume fraction of 0.5 percent may enhance the splitting TS of the material by 19.5 and 31.6 percent, respectively, after 28 days. This is primarily due to the bridging action of the three oriented dispersed fibers across fractures, which is what successfully restrains the proliferation of micro-cracks in the initial stage. After the specimens have been flexurally cracked, the stress is then transmitted to the bridging fibers. As a result, the formation of macro cracks is slowed down, and the splitting TS is increased [75].

The early splitting TS of concrete is not greatly affected by BFs, however, the later splitting TS is significantly affected. At 28 days, concrete with a 0.05 percent fiber content had the maximum splitting TS (3.74 MPa), 1.36 percent greater than control concrete. The splitting TS of the concrete diminishes as the fiber content rises and falls below that of the control concrete. The splitting TS at 90 days increased by 4.41 percent to 9.35 percent with the addition of 0.05 percent to 0.15 percent BFs to concrete. However, the addition of 0.2 percent BFs reduces the splitting TS by 1.29 percent. The tensile stress of concrete is mostly supported by concrete prior to fracture. Tensile stress is transferred from the matrix to the BFs between cracks once the matrix has cracked. The splitting TS of concrete is enhanced by the redistribution of an internal force, which also decreases the stress concentration factor at the concrete’s microcracks, increases the concrete’s ultimate tensile strain, and prevents crack development and expansion. The overall surface area of the fiber rises, nevertheless, when the fiber content is quite high, necessitating the use of a lot of cement paste. As a result, the binding between the cement paste and the aggregate and the splitting TS are adversely affected [51].

The TS of concrete combined with BFs of 12 mm, 24 mm, and 50 mm in length was examined. The results showed that using 12 mm BFs with a content of 1–2% allowed for the best possible TS of concrete. In addition, BFs reinforced concrete with a content of 1–3 percent has a length of 24 mm and a length of 50 mm, and its TS is significantly boosted by 1.80 times. Although most fiber lengths have excellent TS, there is no difference between employing fiber lengths of 24 mm and 50 mm [76]. The splitting TS showed a good correlation between the resistance of BFs to concrete medium fracture and to this circumstance [32]. According to research, BFs at low dosages (0.05–0.5%) may boost splitting TS by 14–25.5 percent [36]. 

**Figure 10:**
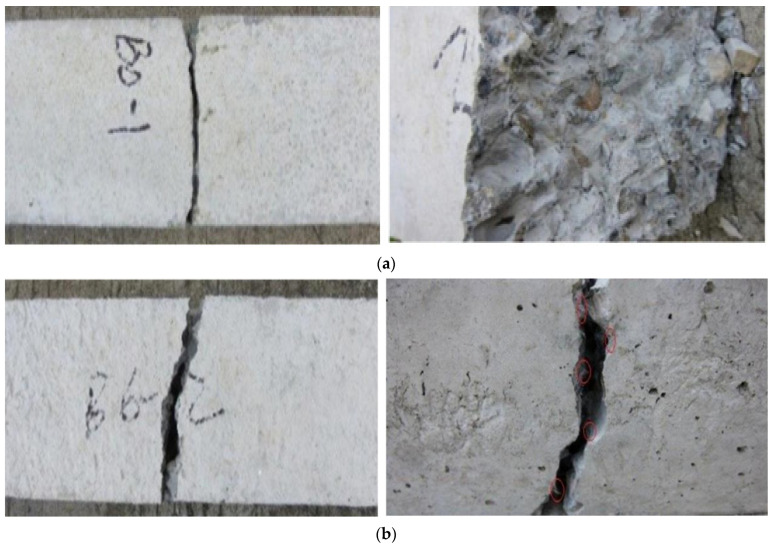
Tensile Failure Mode (**a**) Control and (**b**) Basalt Fiber Reinforced [78].

Throughout the first part of the test, the load continuously increased as the displacement increased. The rising amplitude of the load value showed no evident change when the deformation approached the ultimate displacement. As seen in Figure 10a, the specimen split into two pieces when macroscopic fractures started to emerge in the center of it. The cracking process was very quick, and the portions that formed were smooth and unclouded. During the first loading phase, the load increased linearly as displacement increased. The rate of load increase reduced as the deformation neared its maximum displacement. When macroscopic fractures were found in the center of the specimen, the specimen fractured into two distinct but unequal parts. A lot of tensional fibers were present in the fracture surface of basalt fiber concrete, as shown in Figure 10b, and the fractured sound was lower than in the case of plain concrete. The tensile load was almost nil because the basalt fiber’s elastic modulus was too low to support the load adequately. The fibers at the fracture were shown to be torn off after the fracture when the specimen was stretched further, indicating that the basalt fibers had a strong connection with the concrete.

The best use of the fibers’ bridging function is to stop or slow the spread of fractures in composite materials. Concrete cracking is a multiscale phenomenon that is first manifested as a small microcrack and ultimately grows to a significant macrocrack. According to one study [56], the presence of steel fibers changes the failure mechanism from numerous cracking to a few small, localized fractures. In addition, research [77] found that the presence of fibers considerably enhances the performance of reinforcement during and after cracking.

### 4.3. Flexural Strength (FS)

**Figure 11:**
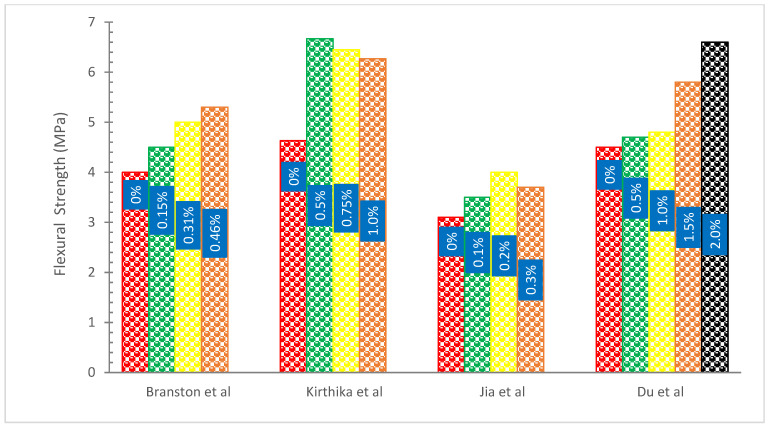
Flexural Strength: Data Source [33,67,68,70].

Figure 11 shows the FS of concrete with the addition of basalt fibers. It can be observed that the addition of BFs increased the FS of concrete. A researcher examined the FS of concrete inclusions with various volumes of BFs. By adding 1% BFs to concrete, it was estimated that the modulus of rupture would drop after seven days [79]. BFs were added to concrete by the researcher, who then examined the beam specimens’ flexural characteristics. It was determined from the results that BFs may significantly increase the FS of concrete beams. Its bridging function may successfully stop a fracture from spreading [80]. The mechanical properties, CS, crack resistance, and FS of concrete blended with 0.1 percent, 0.15 percent, 0.2 percent, 0.25 percent, 0.3 percent, and 0.35 percent of BFs were studied using crushed basalt-reinforced concrete with a length of 30 mm and various amounts of cement. It was discovered that the FS of concrete improves as the concentration of BFs does. FS reaches its peak with a BFs concentration of 3% [81].

In comparison to the control mixture, the flexural strengths of the mixes were significantly boosted by the inclusion of BFs. With varying fiber lengths, there was no discernible difference in the seven-day flexural strengths. These findings suggested that BFs efficiently bonded to the cement matrix regardless of fiber length. Additionally, BF-reinforced composites had a greater increase in FS than glass fiber-reinforced composites when compared to plain mortar. In fact, it was discovered that BF composites (20 mm length) with 0.5 and 1 percent fiber had flexural strengths that were 19 and 16 percent greater than those of fiber mixes (20 mm length) with the same fiber content. In applications requiring reinforcement, BFs seem to be more advantageous than glass fiber. Furthermore, it is evident from comparing the flexural data that the peak load of BF composites occurred at a much greater deflection compared to that of glass fiber composites. This is explained by the fact that BFs have more TS and elongation than glass fibers [35].

Increases in the volume percentage of BFs make the gain in FS more noticeable. As opposed to fiber-reinforced concrete with a fiber volume fraction of 0.3 percent, however, FS shows a modest decrease at 0.5 percent. This might be because high-volume fiber dispersion with a 0.5 percent fraction has proved challenging. It follows that 0.3 percent of the total volume of concrete is the appropriate amount of BFs to admix. Furthermore, FRC with 22 mm BFs has greater FS than fiber-reinforced concrete with 12 mm BFs. The anchoring and bridging effects are greater in longer fibers. In addition, the connection between the fiber and the concrete matrix strengthens, which will aid in the growth of flexural strength. Additionally, it has been shown that plain concrete exhibits brittle behavior. The specimen of plain concrete will split in two when subjected to flexural pressure after a bending fracture appears. However, the fiber actually slows down the crack’s spread, improving the specimen’s mechanical behavior and toughness [36].

The flexure and serviceability behavior of concrete reinforced with ribbed basalt rebars were described by research. Rebars with diameters 8, 12, and 16 mm were used. The four-point bending test was used to evaluate the beams. Due to the axial stiffness of the flexural reinforcement being comparable to that of other bars, rebar with smaller diameters had better bonding with the concrete with respect to bar diameter and spacing. As a result, its behavior was determined to be better under cracking [82]. According to research, BF content rose as FS increased. The authors used varying BF contents and put them in the lab with various concrete beams. It was suggested that increasing the amount of BFs in concrete would increase the modulus of rupture [83].

**Figure 12:**
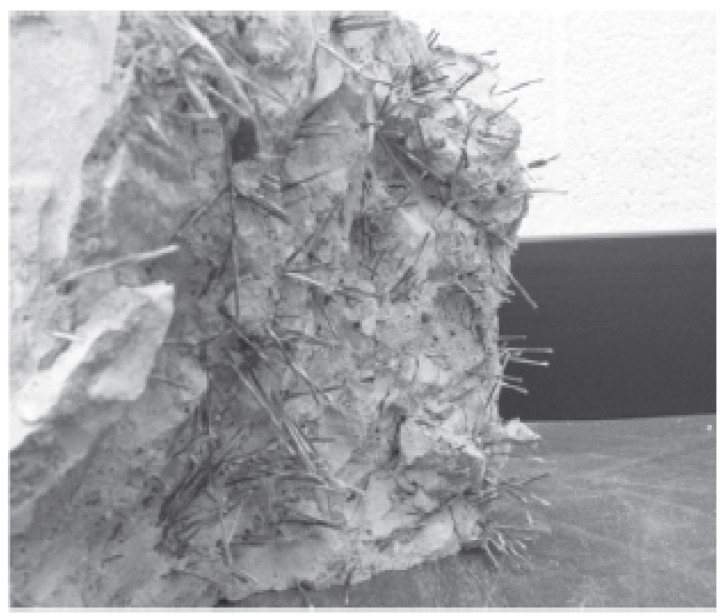
Fracture Surface of Concrete After Flexural Strength [38] with Elsevier permission.

The poor post-crack performance of BFs was not unexpected considering the absence of discernible fibers in the cracked cross-section, as illustrated in Figure 12. It was assumed that the chopped fibers had some degree of degradation since even at the greatest doses, BFs were not visible to the naked eye. In any case, the BFs raised the first-peak stress and so had some positive effects. Most of the time, BF specimens were found to have lower first-peak stress deflection than PC ones. This would imply that when the fiber dose is increased, the action of the BFs results in an increase in strength and modulus of elasticity. Furthermore, compared to fibers of length 36 mm, this rise is larger with fibers of length 50 mm.

**Figure 13:**
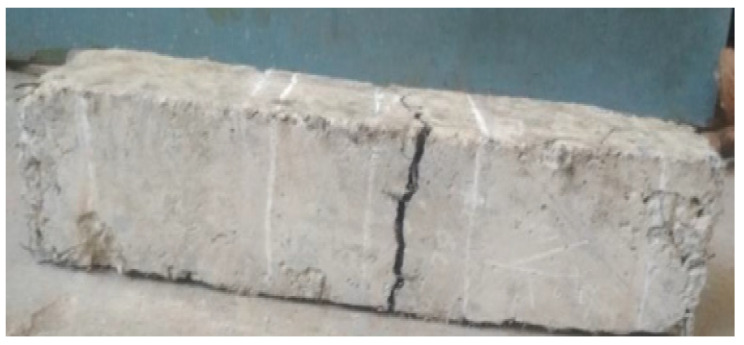
Failure of 1.5% Basalt Fiber Reinforced Concrete [50] with permission of Elsevier.

In contrast, basalt FRC prisms demonstrated ductile failure upon crack development, as seen in Figure 13. The improvement in ductility and fracture resistance was shown by the rise in elasticity. The plane concrete samples were abruptly split into two pieces when a fracture appeared while performing a modulus of rupture test. Because BFs and the concrete matrix were loaded first, the BF beam samples had a higher modulus of rupture than reference concrete beam samples. Later, BFs served as a link between the concrete matrix and the increased load level. As seen in Figure 13, BFs prism samples exhibit wider cracks than typical concrete prism samples. The quantity of fibers and dispersion fibers affects the BFs concrete’s modulus of rupture.

**Figure 14:**
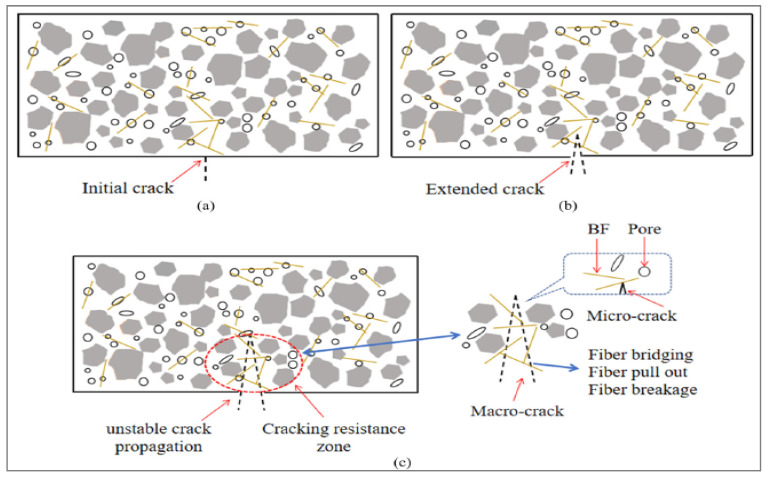
Flexural Failure Process of Basalt Fiber Reinforced Concrete: (**a**) Crack Initiation, (**b**) Crack Propagation, and (**c**) Toughening and Cracking [73].

Figure 14 examines the impact of BFs on the pores and cracks in recycled aggregate concrete, as well as the distribution of BFs in the cement matrix. It depicts the whole process of BFs reinforced recycled aggregate concrete from crack appearance to failure. The fiber may also be thought of as a thin “steel bar” inside recycled aggregate concrete, which directly mimics the fracture resistance mechanism of BFs.

### 4.4. Modulus of Elasticity (MOE)

**Figure 15:**
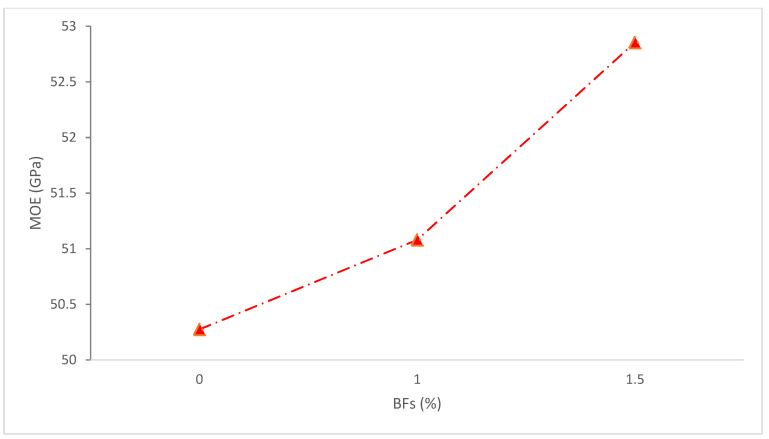
Elastic Modulus: Data Source [84].

Figure 15 shows the MOE of concrete with the addition of basalt fibers. It can be observed that the addition of BFs increased the MOE of concrete. Research demonstrates that the quantity of fiber raises the elastic modulus of concrete, however, the increase is not significant. In comparison to the control, the elastic modulus of the concrete sample rose by 3 and 5.1 percent, respectively, when 1.0 and 1.5 percent of fiber were utilized. Thus, the results demonstrate that the BFs do not significantly enhance the MOE [84]. According to one study, the CS of concrete and MOE are interrelated. In relation to the increase in fiber volume, the findings for CS show a small improvement. The results of the MOE similarly demonstrate no appreciable change in the MOE caused by the insertion of fiber. So, it can be concluded that the elastic modulus is unaffected by the inclusion of BFs [69].

In contrast, an experiment using different ratios of 0.1–0.3% BFs was claimed to considerably boost the MOE. It was discovered that fibers addition 23% increase elastic modulus of concrete compared to the reference concrete [85]. The effect of adding 0.06 percent, 0.2 percent, and 0.8 percent of BFs to the stress and strain curve of concrete was examined by a researcher, who discovered that adding 0.2 percent of BFs may offer the maximum value of stress and strain resistance [86]. According to research, adding BFs to self-compacting concrete that is 6, 12, 24, and 36 mm in length may dramatically raise the MOE [87]. The paper suggests more research into the elastic modulus of BFs reinforced concrete.

### 4.5. Impact Resistance (IR)

**Figure 16:**
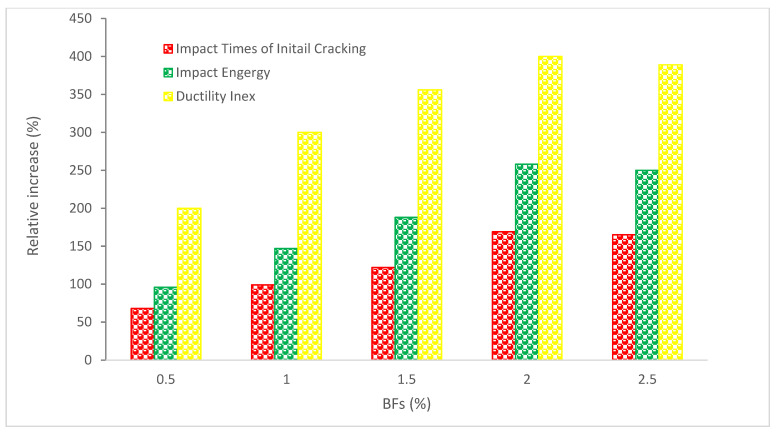
Impact Resistance Behaviors: Data Source [88].

Figure 16 shows the IR of concrete with the addition of basalt fibers. It can be observed that the addition of BFs increased the IR of concrete the test findings show that the BFs blend greatly increases the concrete’s stress resistance. Ordinary concrete is fragile, thus after its first break, a fissure swiftly travels across the cross-section. As a result, there is little difference between the initial-crack impact cycle number and the fracture impact cycle number. The initial-crack impact cycle number of the BF-reinforced concrete rises by 60% to 170%, and the impact energy increases by 90% to 260%.

The ductility index is crucial to the safety of concrete buildings because it measures the energy consumption and deformation capability of concrete when its matrix splits. The ductility index of BF-reinforced concrete rises by a factor of 24 as indicated in Figure 16. Regardless of the beginning crack cycle number, impact energy, or ductility index, a decreasing trend was shown in the experiment after a certain BF dose was reached. In this way, a desirable range of fiber content is established.

Ordinary concrete cracks when subjected to impact loads, while BF-reinforced concrete is slightly fractured but not broken, demonstrating an adequate level of durability. Concrete deterioration is a process in which fractures progressively widen and grow. The fiber number per unit volume in concrete is high after the addition of fiber, which is fine and greater than the surface area, and a homogeneous network system with a three-dimensional disorderly distribution of fiber is produced. This method efficiently slows down the crack’s quick growth and effectively minimizes the damage to the concrete by absorbing kinetic energy while the concrete is being struck. This system does this by reducing the stress concentration of the fracture tip within the concrete. Therefore, an increase in toughness under impact loads is the most important effect of BFs on the concrete matrix.

According to Manibalan and Baskar [89], the energy input required to cause the visibility of the first crack increased by 120 percent, 130 percent, 150 percent, and 124 percent, respectively, in M40 concrete when BFs were added. The energy required to cause the failure of the concrete specimen increased by 108 percent, 125 percent, 142 percent, and 112 percent over the plain concrete specimen. Similar results were obtained for dosages of 0.3 percent, 0.6 percent, 0.9 percent, and 1.0 percent of BFs in M50 concrete, with increases in the energy needed to initiate the first crack of 121 percent, 134 percent, 150 percent, and 131 percent, respectively, and the energy needed to cause specimen failure of 125 percent, 137 percent, 154 percent, and 139 percent, respectively, over the plain concrete specimen. As a result, it was shown that both in the first crack stage and the failure stage, increasing the volume percentage of BFs greatly increases the impact energy of concrete by up to 0.9 percent. This demonstrates how the BFs function as a reliable fracture arrestor in fiber-reinforced concrete when an impact load is present. As a result, as compared to fiber-reinforced concrete, which has greater ductile qualities, plain concrete fails because of its brittle nature.

**Figure 17:**
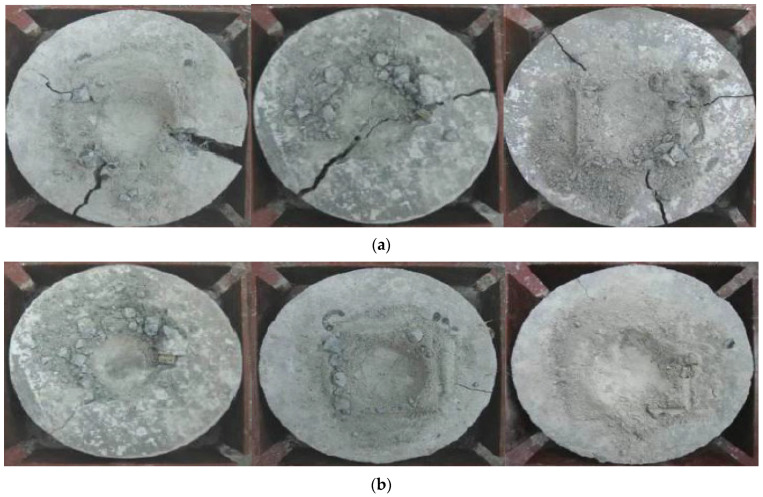
Failure Modes (Impact Resistance): (**a**) Control and (**b**) Basalt Fibers Reinforced Concrete [88].

The fracture extension in the BF-reinforced concrete is delayed by the bridging action in the crack between the fibers, which starts after the cracks develop. In comparison to the standard concrete test blocks, the BFs, which are removed to use a particular amount of energy, result in various alterations in the form of destruction. After cleft, the failure pattern of BF-reinforced concrete is destroyed without pieces. Only many fractures and molting are visible, while the original concrete’s integrity is still intact. As a result, the mixed fiber transforms the brittle failure pattern of concrete into a ductile failure pattern. Figure 17a depicts the typical conventional concrete failure pattern and Figure 17b, depicts the failure pattern of basalt-fiber reinforced concrete.

### 4.6. Toughness 

**Figure 18:**
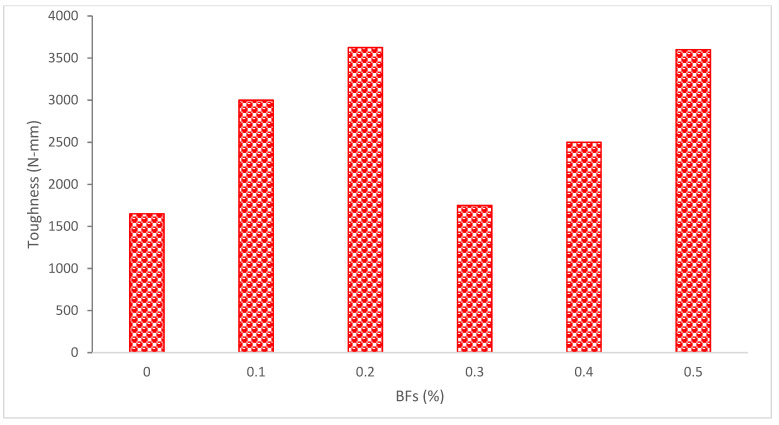
Toughness of Basalt Fibers Reinforced Concrete: Data Source [61].

The impacting behavior and damage progression of BF-reinforced concrete were presented in Figure 18. The outcome demonstrated that the impact toughness of concrete had clearly improved as a consequence of the inclusion of BFs [90]. The bridging action of the fibers may significantly increase toughness and ductility, preventing the rapid brittle fracture that occurs after a peak load in plain concrete [36].

According to research [61], adding more BFs led to an increase in toughness of between 6 and 119 percent when compared to a control mixture without fiber. The toughest combination was M0.5, which had 0.5 percent fibers, with a hardness of 4125 N-mm, whereas M0.0, which contained 0 percent fibers, had a toughness of 1650 N-mm. These findings imply that the ideal fiber level is between 0.2% and 0.5%. One of the key factors in increasing toughness is the quantity of fibers that cross the fracture surface. The ideal fiber content, therefore, is between 0.2 percent and 0.5 percent from the standpoint of toughness. According to research, adding more BFs reduced the mechanical qualities of the concrete even while doing so enhanced the toughness and energy absorption of the concrete. This was due to uneven fiber dispersion. However, the behaviors of concrete were enhanced by the use of admixtures [91]. Research has shown that when the loading rate rises, the toughness increases. The volume portion of 0.2 percent once again demonstrates its ability to withstand impact toughness for its ductility relative to other specimens [92].

According to research, regardless of testing age, it is clear from comparing the toughness data that both peak load toughness and toughness at deflection of 2.5 mm rose exponentially as BFs content climbed from 0 to 1.5 percent. To be more specific, adding 0.5 percent, 1 percent, and 1.5 percent BFs to plain mortar increased its 28-day toughness at a deflection of 2.5 mm by 4, 13, and 33 times, respectively (0.176 KN.mm) [35]. As previously mentioned, more fibers are available for the crack bridging action, which accounts for the improved toughness of composites with larger fiber contents [74]. The research concludes that in plain concrete, after the peak load, the load of unreinforced concrete quickly decreases with an increase in deflection, but the load of BF-reinforced concrete decreases gradually, exhibiting superior toughness performance. The best capability for toughness development is shown by BFs with a length of 22 mm. On the other hand, it is also noted that all of the fiber concrete beams have cracks that are more closely spaced and, as a result, have smaller cracks than plain beams [36].

## 5. Conclusions 

A comprehensive summary and analysis of recent research that is relevant to the mechanical characteristics and failure mechanisms of basalt-fiber-reinforced concrete is given in this paper. The following findings are reached, in consideration of the in-depth discussions presented above.

The flowability of concrete declined with the addition of BFs due to the increased surface area, which required more water and pastes for lubrication. With the inclusion of BFs, a decrease in unit weight and an increase in air content were also seen. As with other forms of fiber, the inclusion of BFs boosted the strength qualities of concrete up to a certain level. However, the ideal BF concentrations are crucial since a greater dosage has a negative impact on strength qualities. Nevertheless, based on factors such as mix design and fiber length, many studies suggest varying ideal BF percentages. However, the normal range of ideal BF percentages is from 0.5 to 1.5 percent. In addition, it can be shown that BFs have a less increased compressive capacity than flexural or tensile capacity. The inclusion of BFs also transforms the brittle character of concrete into a ductile failure, which ensures safety by delivering deformation (warning) prior to failure, according to research on failure modes.

## 6. Recommendations

Research suggests that BFs have not significantly increased the compressive strength of concrete. To enhance the compressive capacity of basalt-fiber-reinforced concrete, further research adding pozzolanic materials to basalt-fiber-reinforced concrete is required. The data on MOE of concrete with the addition of BFs is unclear since some publications claimed that BFs have no effect on MOE while others claimed that MOE has significantly improved. Consequently, a detailed assessment is needed in this area. Due to a lack of flowability, greater dosages of BFs result in lower strength properties. This study, therefore, recommends a thorough examination of various doses of plasticizers at a higher dosage of BFs. Finally, this study compiles data on the fresh characteristics, strengths, and failure mechanisms of basalt-fiber-reinforced concrete. The review also recommends a compressive review of durability aspects, thermal properties, and microstructure analysis of basalt-fiber-reinforced concrete.

## Figures and Tables

**Table 1:** Physical Properties BFs.

Reference	[31]	[32]	[33]	[34]	[35]
Specific gravity	-	-	-	2.65	-
Water Absorption (%)	-	3.60	-	-	-
Density (g/cm^3^)	2.75	2.70	2.68	-	2.64
Tensile Strength (MPa)	4840	3200–4800	2800	4150–4800	4200
Elastic modulus (GPa)	89	90	-	100–110	98
Elongation (%)	3.15	-	4.0	-	3.05

**Table 2:** Summary of Strength Properties of Concrete with BFs.

Ref	BFs (%)	Length (mm)	Optimum (%)	W/C	Slump Flow	Days	Compression Strength (%)	Tensile Strength (%)	Flexure Strength (%)	Remarks
[35]	0 to 1.25	20	1.25		-	7 28	13.7 16.1	111.5 114.8	-	Improved
[68]	0 to 4.6	36	0.31		-	28	5.2	-	25	Improved
[67]	0 to 1.0	24	0.50	0.45	-	7 28	20.6 31.5	- 42.7	- 44.06	Improved
[36]	0 to 0.5	12	0.1	0.60	Decline	7 28 90	8.0 4.6 0.0	-	14.6 8.00 1.2	Improved
[63]	0 to 3.0	6	1.0	0.50	-	7 28	33 6.0	40.0 22.2	-	Improved
[69]	0 to 3	25	2.0	0.40	-	28	3.1	5.0	-	Improved
[43]	0 to 0.2	12	0.20	0.22	-	28	7.21	38.7	15.3	Improved
[50]	0 to 2.0	18	1.5	0.48	Decline	7 28	15 2.9	17.5 23.3	-	Improved
[51]	0 to 2.0	18	5.0	0.34	-	7 28 90	10.47 9.8 17.13	1.99 1.35 9.35	-	Improved
[70]	0 to 0.3	-	0.2	0.38	-	28	7.5	22.2	29.0	Improved
[71]	0 to 1.5	45	0.5	0.44	-	28	5.1	-	8.4	Improved
[31]	0 to 1.5	18	1.0	0.66	Decline	28	4.2	13.5	33.4	Improved
[32]	0 to 1.0	-	0.25	0.50	-	28	9.7	2.3	-	Improved
[33]	0 to 2.0	12	2.0	0.70	-	28 56	4.3 1.8	16.0 9.6	37.7 26.9	Improved
[72]	0 to 0.60	18	0.36	0.43	-	28	8.16	-	22.36	Improved
[34]	0 to 2.0	12	1.5	0.71	-	28	7.7	69.0	39.6	Improved

## Data Availability

All the data available in main text.
